# Development of a diverse set of standard short forms based on the EORTC CAT Core item banks

**DOI:** 10.1007/s11136-023-03373-6

**Published:** 2023-02-28

**Authors:** Morten Aa. Petersen, Hugo Vachon, Mogens Groenvold, the European Organisation for Research and Treatment of Cancer (EORTC) Quality of Life Group

**Affiliations:** 1 Palliative Care Research Unit, Department of Geriatrics and Palliative Medicine GP, Bispebjerg & Frederiksberg Hospital, University of Copenhagen, Bispebjerg Bakke 23B, 2400 Copenhagen, NV Denmark; 2 Quality of Life Department, European Organization for Research and Treatment of Cancer, Brussels, Belgium; 3 Department of Public Health, University of Copenhagen, Copenhagen, Denmark

**Keywords:** EORTC QLQ-C30, Item bank, Short form, IRT

## Abstract

**Purpose:**

The European Organisation for Research and Treatment of Cancer (EORTC) Quality of Life Group has developed item banks covering the 14 domains of the EORTC QLQ-C30 quality of life questionnaire. These allow for dynamic assessment and for forming population/study specific static short forms. To simplify selection of relevant short forms, we here present a portfolio of standard short forms with measurement properties optimized for different populations.

**Methods:**

For each domain, a brief and a long version were constructed for each of three populations having mild, moderate, and severe symptoms, respectively. The most informative items were prioritised while also taking content into consideration. All short forms included at least one QLQ-C30 item. The measurement precision/power of the short forms was compared to the corresponding QLQ-C30 scales using simulations.

**Results:**

In total, 84 short forms were constructed. The brief versions included 3–5 items each, the long versions 5–9 items. Estimated sample size savings using the suggested short forms while maintaining the same power as with the QLQ-C30 ranged 3–50% across domains with median savings of 19% (brief versions) and 28% (long versions), respectively.

**Conclusion:**

The suggested short forms allow for simple selection of items particularly relevant for patients with mild, moderate, or severe symptoms, respectively. They facilitate the use of smaller samples without loss of power compared to the QLQ-C30 scales. The suggested short forms may be used as they are or adapted to the specific aims of individual studies/settings.

## Plain English summary

The European cancer research organization, EORTC, has developed 14 sets of questions assessing symptoms and problems particularly common in cancer patients and relevant in cancer research like fatigue, pain, and physical functioning problems. These sets can be used to select questions for questionnaires customized for specific purposes, e.g., a clinical trial of a new cancer treatment. Such customized questionnaires will be more relevant for the patients and provide more precise assessment of their symptoms and problems. However, choosing the best set of questions for a specific purpose may be challenging. Therefore, the EORTC has developed a collection of predesigned questionnaires optimized for different patients and purposes. These will simplify the selection of relevant questionnaires thereby in a simple way hopefully improve the assessment of cancer patients’ symptoms and problems.

## Introduction

Measures reporting on a patient’s health condition based on direct input from the patient are termed patient reported outcome (PRO) measures (PROMs) [1]. Over the last decades PROMs have become an integrated part of most clinical studies and there has been an increasing interest for using PROMs in clinical practice. PROMs may be a useful tool in patient-centred care as they, among other things, can improve patient-clinician communication and clinician awareness of symptoms [2, 3]. The most commonly used PROMs are static, standardised questionnaires. Such standardised questionnaires ensure comparability across studies/patients as the same set of items is used in all instances. However, one size may not always fit all, i.e., the standardised measure may not fit optimally to the specific needs of a study/setting. For instance, if assessing patients expected to have poor physical functioning (PF), it is preferable to use items with high sensitivity at lower levels of PF rather than a standardised measure with items developed to capture the entire PF spectrum. Ideally, a PROM should be adapted to the specific study while retaining the comparability of scores. This is feasible when PROMs based on item banks, i.e., repositories of item response theory (IRT) calibrated items, are used. IRT refers to a family of statistical models used to characterise the psychometric properties of a set of questionnaire items [4, 5]. Calibration of items to an IRT model (i.e., estimation of the IRT item parameters) permits comparison of scores based on any subset of items from the bank, as these will all be on the same metric. This is fully utilised in computerized adaptive tests (CATs) where item selection is tailored to the individual based on responses to prior items. By presenting the most informative items to each patient, measurement precision is optimised [6]. CAT requires ‘live’ computations and hence, can only be conducted electronically with access to suitable CAT-software. Therefore, CAT assessment may not be feasible in all studies or clinical settings. Further, some patients may not be able to complete an online questionnaire or may not be comfortable doing so, preferring an ‘old fashion’ paper questionnaire. Hence, also when CAT is feasible in a study/clinical setting it may be necessary to supplement this with paper questionnaires to accommodate all patients. In such cases so-called short forms may be used. A short form is a static measure consisting of items selected from an item bank to optimize measurement for a specific purpose and/or population. Short forms may be administered either on paper or electronically. Scores from CATs and short forms based on the same item bank are directly comparable.

The European Organization for Research and Treatment of Cancer (EORTC) Quality of Life Group (QLG) was formed in 1980. Currently (2022), the group includes more than 200 active members covering a broad range of professions, including both clinicians and research methodologists, and representing more than 15 countries (see https://qol.eortc.org/quality-of-life-group/ for more details). The group’s core quality of life questionnaire, the EORTC QLQ-C30 [7, 8], is one of the most widely used PROMs for the assessment of Health-Related Quality of Life (HRQoL) in cancer research and clinical practice [8]. To improve measurement of the 14 functional and symptom HRQoL domains covered by the EORTC QLQ-C30, the EORTC QLG developed the EORTC CAT Core instrument [9, 10]. The EORTC CAT Core includes an item bank for each of these HRQoL domains allowing for CAT and short form measurement. For users only familiar with standardised questionnaires like the EORTC QLQ-C30, short form assessment, which resembles assessment with traditional, static questionnaires, may often seem simpler and more manageable than dynamic CAT assessment. Still, assembling the optimal short form for a specific purpose may not be a simple task. Which and how many items should be selected? What are the psychometric implications of choosing one short form over another? Having a collection of short forms with known measurement properties, optimised for different purposes may greatly simplify the task of selecting an appropriate short form. Such ‘standard’ short forms may be used as they are or serve as a starting point for further work towards the assembly of a study specific short form.

In this paper, we introduce a general approach for assembling an appropriate short form for a specific purpose and population and present a collection of standard short forms based on the EORTC CAT Core item banks with measurement properties optimised for different populations.

## Methods

### The EORTC CAT Core

The EORTC CAT Core includes 14 item banks covering the five functional and the nine symptom domains of the EORTC QLQ-C30 questionnaire. The item banks include 7–34 items each with a total of 260 items [9]. All item banks include the QLQ-C30 item(s), supplemented with additional items, covering the same aspects of a particular HRQoL domain as the QLQ-C30 item(s) and using the same timeframe and response options. This ensures measurement within a well-established conceptual framework and maximum backward compatibility with QLQ-C30 while enabling more flexible and precise measurement. The superior measurement properties of the EORTC CAT Core have been confirmed in independent validation studies [11, 12]. All measures based on the EORTC CAT Core are scored on a so-called T-score metric, scaled so that the European general population has a mean of 50 and a standard deviation of 10 [13]. This means that a score > 50 for a functional domain reflects better functioning than the average European general population while for a symptom domain a score > 50 reflects more symptoms than the average general population.

### Short form selection procedure

The aim was to have a collection of standard short forms relevant for different populations and purposes, i.e., a brief form for quick assessment and a longer form for more precise/in-depth measurement, for each of three patient populations with different levels of symptoms for each HRQoL domain. That is, six short forms for each domain and a total of 14*6=84 short forms were developed.

The three target populations, termed mild, moderate, and severe, for each domain were assumed to be normally distributed with mean and standard deviation (SD) based on the QLQ-C30 items. The ‘mild symptom’ population was defined to have a mean corresponding to the average T-score obtained if answering ‘not at all’ or ‘a little’, respectively, to the QLQ-C30 items of the domain. Hence, this population represented patients typically having ‘a little’ or less symptoms. Similarly, the ‘moderate symptom ‘ population had a mean corresponding to the average T-score obtained if answering ‘a little’ or ‘quite a bit’, while the ‘severe symptom’ population’s mean corresponded to the average T-score obtained if answering ‘quite a bit’ or ‘very much’ to the QLQ-C30 items. For each mild population, the SD was chosen so 50% of the population had scores between the scores obtained if answering ‘not at all’ or ‘a little’, respectively, to the QLQ-C30 items of the domain. The SDs for the moderate and severe populations were defined similarly. Selection of items primarily focused on the central interval [mean-SD, mean + SD] where about two-thirds of the population’s scores are expected. As an example, answering ‘a little’ to the three QLQ-C30 fatigue items results in a T-score estimate of 54 while answering ‘quite a bit’ results in a score of 64. Hence, the moderate population for fatigue was defined to have mean = 59 ((54 + 64)/2), SD = 7 (so 50% of patients have scores between 54 and 64) and the ‘interval of focus’ was 52–66 (59–7 to 59 + 7).

To assess how informative each item was for a given population, the average item information across the interval [mean-SD, mean + SD], weighted by the population distribution, was calculated (this is similar to the maximum posterior weighted information criterion, MPWI, with the population distribution replacing the posterior distribution [14]). The information may be used to calculate average reliability of the items, however, the principal use of these item information values was relative to each other for the selection of items. That is, items with higher information values provide more information about the population of focus, and hence, other things being equal, will be preferable to include in a short form. All short forms were required to include the QLQ-C30 item for HRQoL domains with only one QLQ-C30 item and at least two QLQ-C30 items for HRQoL domains with multiple QLQ-C30 items. In addition, if an item bank covered several content categories (e.g., the fatigue item bank included items on physical and general fatigue), short forms were required to include at least one item from each category to ensure appropriate content balance. The length of each short form was chosen individually and was a balancing of length and precision. For the brief versions, efficiency was given priority while precision was priority for the long versions. As experience from developing the EORTC CAT Core indicate that asking less than three items often provides low precision and asking more than 10 items rarely provides more than trivial additional precision, we expected the brief forms to have 3–6 items and the long forms to have 5–10 items. The long forms consisted of the items from the brief version plus additional items for increased measurement precision.

### Evaluation of short form measurement precision

Using Monte Carlo simulation, we evaluated the relative measurement precision of the short forms compared to the QLQ-C30 scales. The QLQ-C30 scales were scored following the official scoring of the questionnaire, i.e., the scales were sum scores based on 1–5 items each depending on the domain [15]. For each target population and short form, 1000 simulations were conducted. In each simulation two groups of true domain scores were sampled, each of random size between 50 and 250 representing common group sizes in HRQoL studies. One group was sampled from the target population and the other from a population normally distributed with the same SD as the target population but with a randomly selected mean, which differed from the target mean corresponding to an effect size (standardized mean difference) between 0.2 and 0.5 representing small to medium group differences. Based on the sampled domain scores, item responses were simulated and from these, short form and QLQ-C30 scale scores were calculated. As an example, a simulation for the moderately fatigued population could consist of comparing n = 100 randomly selected ‘individuals’ from the target population with mean = 59 and SD = 7 (N(59, 7^2^)) with another group of n = 100 randomly selected from the population N(56, 7^2^) (resulting in a ‘true’ effect size difference of 0.43). Hence, 100 fatigue scores were sampled randomly from each of the two populations. For each of these ‘true’ fatigue scores the probability of responding ‘not at all’, ‘a little’,’quite a bit’, and ‘very much’ were calculated for each item and based on these response probabilities a random response was selected. In this way a set of item responses were generated which was used to calculate estimated fatigue scores based on the short forms and the QLQ-C30 items, respectively.

Two-sample t-test statistics for comparing the groups were calculated for the short form and QLQ-C30 scale, respectively. From these t-statistics the relative validity/efficiency of the short form compared to the QLQ-C30 scale was estimated as the ratio of the short form t-statistic to the QLQ-C30 scale t-statistic: t(short form)/t(QLQ-C30) [16, 17]. The relative validity (RV) assesses the relative sensitivity or known groups validity of the short forms compared to the QLQ-C30 scales [16]. An RV > 1 indicates higher sensitivity/known groups validity of the short form. The median relative validity across the 1000 simulations was calculated and from this, the median relative sample size requirement of the short form compared to the QLQ-C30 scale was estimated [17].

All evaluations and simulations were conducted using SAS Enterprise Guide 7.15.

## Results

To illustrate the selection process applied for all short forms, the selection of short forms for a moderately fatigued population (mean = 59, SD = 7) will be used as an example. The 34 items available in the fatigue item bank are listed in Table 1. The EORTC QLQ-C30 fatigue scale includes three items (items 17, 22, and 27). The item bank (and scale) covers two content categories, physical and general fatigue [18]. According to our selection criteria, the short forms must comprise at least two of the QLQ-C30 items, with at least one item covering general fatigue and another item capturing physical fatigue. The two most informative QLQ-C30 items for the moderately fatigued population are item17 ‘Were you tired?’ and item22 ‘Have you felt weak?’ (see Table 1). Item17 covers general fatigue while item22 primarily concerns physical fatigue. Hence, by including the two items, both the QLQ-C30 and content coverage criteria have been fulfilled. Items 13, 16, 24, and 28 provide the most information on average for this population and they provide similar levels of information (between 0.039 and 0.041, see Table 1). Item13 ‘Have you felt physically exhausted?’ and item16 ‘Have you felt exhausted?’ seem too similar in content to include both in a short form. Item16 is the simplest and most general and may therefore be preferable. Hence, the suggested brief standard short form consists of the five items 16, 17, 22, 24, and 28.

**Table 1:** Average information of the fatigue items weighted following the population density function of the moderately fatigued. Items included in the brief and long standard short forms are marked (√)

Item	Info	Item text	Brief	Long
Item28	0.041	Have you required frequent or long periods of rest?	√	√
Item13	0.041	Have you felt physically exhausted?		
Item16	0.040	Have you felt exhausted?	√	√
Item24	0.039	Have you had a feeling of overwhelming and prolonged lack of energy?	√	√
Item31	0.035	Have you had an extreme need for rest?		
Item25	0.034	Have you had trouble finishing things because you were tired?		√
Item20	0.034	Have you become easily tired?		√
Item11	0.034	Have you been too tired to do even simple things?		√
Item7	0.033	Have you been too tired to do your usual activities?		
Item19	0.032	Have you lacked energy?		
Item30	0.029	Have you become tired from carrying out your duties and responsibilities?		
Item8	0.029	Have you felt drained?		
Item17^*^	0.029	Were you tired?	√	√
Item5	0.028	Have you lacked the energy to do things?		
Item23	0.028	Have you felt worn out?		
Item10	0.028	Have you had trouble starting things because you were tired?		
Item9	0.027	Have you been so exhausted it felt almost impossible to move your body?		
Item14	0.027	Have you found leisure and recreational activities exhausting?		
Item22^*^	0.026	Have you felt weak?	√	√
Item12	0.026	Have you found shopping and doing errands exhausting?		
Item33	0.025	Have you felt tired for a long time after physical activity like taking a long walk?		
Item32	0.024	Have you become exhausted from dressing?		
Item6	0.022	Have you felt slowed down?		
Item34	0.022	Have you become exhausted from taking a shower?		
Item27^*^	0.019	Did you need to rest?		
Item4	0.017	Have you started things without difficulty but got weak as you went on?		
Item21	0.016	Have you had trouble sitting up because you were tired?		
Item26	0.015	Have you become tired from walking up stairs?		
Item2	0.015	Have your muscles felt very tired after physical activity like taking a long walk?		
Item15	0.015	Have you felt weak in your arms or legs?		
Item29	0.013	Have you been too tired to eat?		
Item18	0.013	Have you had to sleep for long periods during daytime?		
Item3	0.010	Have you woken up with a feeling of exhaustion?		
Item1	0.009	Have you been so tired it was difficult keeping your eyes open during daytime?		

^*^EORTC QLQ-C30 items

Concerning the long standard form, items 11, 20, 25, and 31 are the most informative of the remaining fatigue items. They provide almost the same level of average information (0.034–0.035). Having an extreme need for rest (item31) may be similar to requiring frequent or long periods of rest (item28). Hence, from a content point of view it may not be relevant to include item31 in addition to the already included item28. Items 11, 20, and 25 do not overlap significantly in content with the already selected items, thus, these could be added to a long version. Although more items could be added, eight items seem a sensible length with increased precision compared to the brief version (see below). Therefore, the suggested long version consists of the eight items 11, 16, 17, 20, 22, 24, 25, and 28 (see Table 1 for full item texts).

**Fig. 1:**
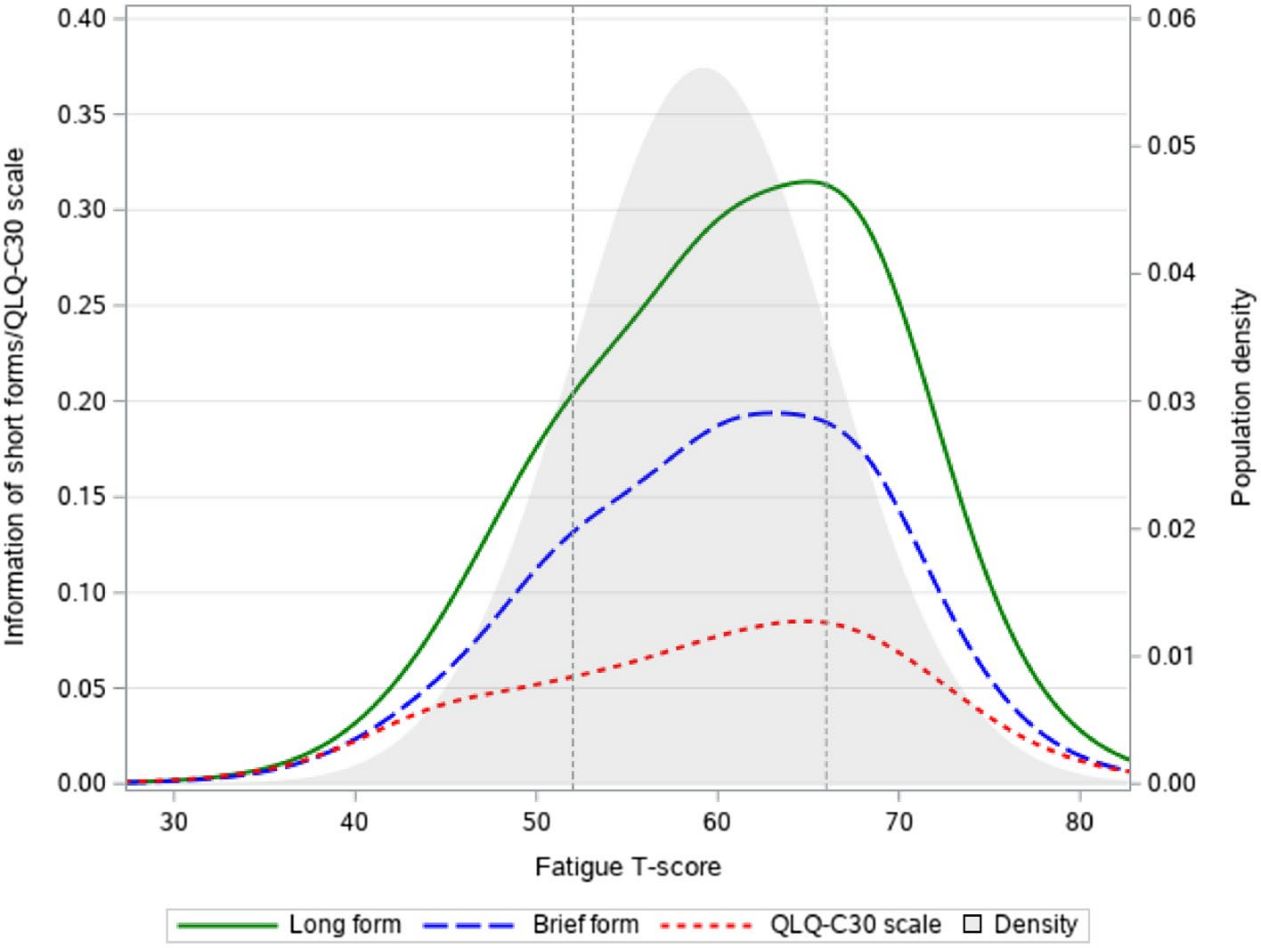
Information functions for the selected fatigue short forms and the QLQ-C30 fatigue scale and the population density function for the moderately fatigued population is shown in grey. Fatigue scores within the vertical dotted lines are of particular focus

The information provided by the suggested short forms (brief and long versions) compared to the QLQ-C30 fatigue scale are shown in Fig. 1. As can be seen, the suggested short forms provide markedly more information, particularly for fatigue levels close to the population mean (say scores in the range 50–70), where most of the population is located. To get an impression of the practical implications for sample size requirements using the suggested short forms compared to using the QLQ-C30 fatigue scale, we simulated the abilities of the short forms to detect group differences compared to the QLQ-C30 scale. These simulations indicated that using the brief short form may reduce sample size requirements by 12% on average while providing the same power as the QLQ-C30 scale. Using the long version, samples may be reduced by 17% (see Table 3). Adding item13 to the long version, the most informative item not included, also results in median savings of 17% (details not shown). Hence, adding more items does not increase power further.

Using similar approaches and arguments for including/excluding items as presented for the fatigue short forms, we developed totally 84 standard EORTC short forms across the 14 domains. The number of items included in each of the standard EORTC short forms are shown in Table 2. The brief versions include 3–5 items each (median = 4 items) while the long versions include 5–9 items (median = 7 items). The median relative validity (RV) and derived sample size savings using the standard short forms compared to using the EORTC QLQ-C30 scales are presented in Table 3. The estimated savings in sample size requirements varied across domains from 3% (brief version for population with severe physical problems) to 50% (long version for population with severe nausea/vomiting). Across domains and populations, the median sample size savings using short forms compared to using the EORTC QLQ-C30 scales was 19% (18–24% for each population) for the brief versions and 28% (24–32%) for the long versions.

**Table 2:** Number of items in each EORTC CAT Core item bank (number of items in the corresponding QLQ-C30 scale) and in each of the standard short forms suggested for populations with mild, moderate, and severe symptoms, respectively

Domain	Item bank	Mild brief	Mild long	Moderate Brief	Moderate long	Severe brief	Severe long
Cognitive functioning	34 (2)	4	8	4	8	4	8
Emotional functioning	24 (4)	5	8	5	9	5	9
Physical functioning	31 (5)	5	9	5	9	5	9
Role functioning	10 (2)	4	7	4	7	4	7
Social functioning	13 (2)	4	7	4	7	4	7
Constipation	10 (1)	3	5	3	6	4	8
Diarrhoea	13 (1)	4	6	3	6	3	7
Dyspnoea	32 (1)	4	7	4	7	4	7
Fatigue	34 (3)	5	8	5	8	5	8
Financial difficulties	9 (1)	3	5	4	6	4	8
Insomnia	8 (1)	3	6	3	6	3	6
Lack of appetite	7 (1)	3	5	3	5	4	6
Nausea & vomiting	19 (2)	4	8	4	8	4	9
Pain	16 (2)	4	8	4	8	5	8
Total across 14 domains	260 (28)	55	97	55	100	58	107

**Table 3:** Estimated median relative validity (RV) and sample size savings (save) using the suggested standard short forms compared to the EORTC QLQ-C30 scales

Domain		Mild brief	Mild long	Moderate Brief	Moderate Long	Severe brief	Severe long
Cognitive functioning	RV Saving	1.11 19%	1.14 22%	1.13 20%	1.19 28%	1.11 19%	1.19 28%
Emotional functioning	RV Saving	1.03 5%	1.07 12%	1.05 9%	1.09 16%	1.06 11%	1.11 19%
Physical functioning	RV Saving	1.10 17%	1.13 20%	1.02 3%	1.06 11%	1.02 3%	1.05 9%
Role functioning	RV Saving	1.10 17%	1.16 25%	1.09 16%	1.18 27%	1.11 19%	1.16 25%
Social functioning	RV Saving	1.05 9%	1.13 20%	1.06 11%	1.14 22%	1.16 25%	1.20 30%
Constipation	RV Saving	1.15 23%	1.21 31%	1.15 23%	1.26 36%	1.26 36%	1.32 41%
Diarrhoea	RV Saving	1.09 16%	1.14 22%	1.16 25%	1.23 33%	1.21 31%	1.29 39%
Dyspnoea	RV Saving	1.27 38%	1.29 39%	1.27 38%	1.35 44%	1.24 34%	1.31 41%
Fatigue	RV Saving	1.08 14%	1.10 17%	1.07 12%	1.10 17%	1.07 12%	1.09 16%
Financial difficulties	RV Saving	1.11 19%	1.18 27%	1.18 27%	1.23 33%	1.25 34%	1.29 39%
Insomnia	RV Saving	1.16 25%	1.24 34%	1.11 19%	1.24 34%	1.09 16%	1.23 33%
Lack of appetite	RV Saving	1.12 19%	1.19 28%	1.16 25%	1.21 31%	1.22 31%	1.28 38%
Nausea & vomiting	RV Saving	1.24 34%	1,34 44%	1.36 45%	1.43 50%	1.31 41%	1.42 48%
Pain	RV Saving	1.03 5%	1.12 19%	1.11 19%	1.17 27%	1.15 23%	1.17 27%
Median across 14 domains	RV Saving	1.11 18%	1.14 24%	1.12 20%	1.20 30%	1.16 24%	1.22 32%

An RV > 1 indicates higher precision of the short form

Further details on the use of EORTC short forms and the EORTC CAT instrument in general may be obtained from the EORTC QLG at https://qol.eortc.org/cat/.

## Discussion

Short forms, i.e., static measures of items selected from an IRT calibrated item bank, may be viewed as an intermediate solution between traditional ‘static’ instruments and dynamic CAT assessment. Compared to traditional questionnaires as the EORTC QLQ-C30 short forms provide increased flexibility to adapt the questionnaire to the specific purpose of a study or clinical setting. From a larger pool the most relevant items can be selected. More items can be included for key domains to increase measurement precision while fewer items can be chosen for less important domains. However, choosing the ‘optimal’ short form for a specific purpose may not be simple. For instance, over 17 billion different short forms can be composed from an item bank with 34 items such as the EORTC fatigue bank. Clearly, not all possible combinations can be evaluated, and one must adopt a simplified strategy for selecting items to construct a short form. Here we have presented a standardized approach for selecting items for short forms. We used this to generate six suggestions for short forms for each of the 14 functional and symptom HRQoL domains covered by the EORTC CAT Core item banks. Each short form is optimized for measurement in one of three populations: patients typically having mild, moderate, or severe symptoms, respectively. As expected, the short forms provide higher measurement precision/lower sample size requirements than the corresponding EORTC QLQ-C30 scales.

The short forms measure the same HRQoL domains as the QLQ-C30 scales, however, as the short forms are IRT scored while the QLQ-C30 scales are sum scored, scores with one instrument cannot be directly compared to scores on the other. To simplify interpretation the short forms (as any EORTC CAT Core measure) are on a T-score metric so that all scores can be interpret relative to the European general population. Thus, since the general population has mean = 50 and SD = 10, a fatigue score of e.g., 55 would indicate that the patient is more fatigued than about 70% of the general population. To ensure simple and correct scoring, the short forms are scored using a simple scoring service/program developed by the EORTC QLG. Alternatively, scoring tables can be provided by the EORTC QLG.

Selecting the best short form requires knowledge about (or at least a qualified guess of) the symptom level of the target population. Reflecting the way, we defined target populations, the ‘mild population’ has a mean score of 17 on the QLQ-C30 sum scale for all symptom domains and a mean sum score of 83 for the functional domains. Similarly, the ‘moderate population’ has a mean sum score of 50 and the severe population has a mean sum score of 83 (symptom scales) and 17 (function scales), respectively. Investigators selecting short forms may look into historical data using the QLQ-C30 (e.g., the EORTC QLG reference values [19]). For example, if previous studies for a given patient population have found mean QLQ-C30 scores in the range 35–65 for fatigue, then the short forms developed for moderately fatigued patients are likely the best choice. Note that, particularly for the item banks with fewer items there may be considerable overlap between the short forms for the different populations. As an extreme example the short forms for patients having mild or moderate lack of appetite, respectively, include the same items. In such cases foreknowledge of the symptom level is of less importance. In other cases, there may be considerable differences between short forms, making such foreknowledge more important. For example, the short forms for patients having mild or severe physical problems, respectively, do not have any items in common.

Sometimes information regarding anticipated symptom level is not available or an instrument with broad coverage is needed. In such cases one could combine the items from the brief versions for the three symptom-level populations. For example, for fatigue this would result in a 10-item short form (as some items are included in two or three of the brief versions). This illustrates that the standard short forms suggested here should not be viewed as the only suitable short forms. On the contrary, they should mainly be viewed as ‘sensible starting points’ for the construction of relevant short forms. In some cases, they are just what is needed, in others, they may benefit from small adaptations, deleting an item, adding another, etc., to adapt them to the specific needs of a study (any adaptations should be done in agreement with the EORTC QLG).

A typical application could be to use long versions for a few key domains, brief versions for domains of secondary interest and just 1–2 items/domain to cover remaining domains. For example, having one primary outcome and 3–4 secondary outcomes, the 14 domains may typically be covered sufficiently with less than 40 items. Constructing short forms is always a balance between measurement precision and response burden—enough items should be included to obtain the necessary precision while at the same time patients should not be burdened with answering unnecessary items. When judging response burden, it may be useful to note that previous validation of the EORTC CAT Core found that 90% of patients used less than 17 s answering each item [10].

In CAT assessment the most common item selection strategy is to select items based on level of information [6]. In each step of the CAT the most informative item is selected, thereby optimizing the measurement precision. We applied a similar principle for the short form item selection, giving priority to the items providing the most information for the population of focus. Content was also considered when selecting items by requiring that all content categories of a given HRQoL domain were covered and generally avoiding items of highly similar content. Item information combined with content considerations have also been used to construct e.g., PROMIS physical functioning and fatigue short forms [20, 21].

Although information may be a common criterion for selecting items for IRT-based short forms, it may be used in different ways. We prioritized items with high average information resulting in primarily selecting items being informative where the majority of the population of focus is located. Alternatively, one could select items being informative at different locations across the score continuum to obtain a more even level of information/precision across a broader range of scores. This could be particularly relevant if measuring in a highly heterogeneous population. Alternatively, if the aim is classification in e.g., cases (requiring treatment) and non-cases, one could select items being particularly informative around the cut score for case/non-case, thereby increasing the chance of true classification. Construction of such customized short forms can be conducted in close collaboration with the EORTC QLG drawing on the group’s experience with PRO development and knowledge about the psychometric properties of the items.

Information functions (and summaries of these) are highly useful for assessing the measurement value and precision of individual items and sets of items. However, it may not be simple to convert the provided information into practical impact. For example, adding an item will increase the total information obtained with a short form, but will this reduce the required sample size for a study and to what extend? Such knowledge may be valuable when deciding on the number of items to include in a short form. To assist in judging the ‘practical impact’ of choosing a short form, we have simulated the expected relative sample size savings of using the short forms compared to using the QLQ-C30 scales. Given that most of the short forms include more items than the corresponding QLQ-C30 scales (the brief versions include two more items and the long five more on average) it is not surprising that the short forms provide sample size savings. Nevertheless, the simulation results provide useful insight about the possible savings when deciding on the most appropriate measure for a given purpose. As the simulations assessed a limited set of cross-sectional scenarios only (comparing two groups of size 50–250 with effect size difference of 0.2–0.5), the findings may not generalize to other settings, e.g., for assessing changes over time or differences in populations deviating markedly from the populations investigated here. Future research could expand on the current simulations to such settings and in general assess in more details the psychometric properties of the short forms. It should be noted that the provided estimated savings are averages (medians) and variation across individual studies should be expected. Nonetheless, the estimated savings provide a useful ‘practical’ addition to information functions when choosing a short form.

## Conclusion

Based on item information and content considerations we have developed 84 standard short forms, i.e., six short forms for each of the 14 domains covered by the EORTC CAT Core item banks. The short forms allow for simple selection of items particularly relevant for populations with predominantly mild, moderate, and severe symptoms, respectively. Although variation across domains were observed, simulations indicated that the short forms facilitate the use of smaller samples, 19–28% on average, without loss of power compared to using the EORTC QLQ-C30 scales. The suggested EORTC CAT Core short forms may be used as they are or adapted to the specific aims of individual studies/settings. For further information on the use of EORTC short forms, please visit https://qol.eortc.org/cat/.

## Data Availability

The data are not publicly available.
